# 

**Published:** 1892-03

**Authors:** 


MARCH, 1892.

*Vol. X.* **No. 35.**

THE

### BRISTOL MEDICO-CHIRURGICAL JOURNAL

LIBRARY
Indexed
S. G. O.

530

A Quarterly Journal of the Medical Sciences for the West of England and South Wales

*PUBLISHED UNDER THE AUSPICES OF*

*THE BRISTOL MEDICO-CHIRURGICAL SOCIETY.*

EDITOR:

R. SHINGLETON SMITH, M.D., B.Sc.

ASSOCIATED WITH

BARCLAY J. BARON, M.B., C.M.

J. MICHELL CLARKE, M.B., M.A.

F. R. CROSS, M.B.

W. H. HARSANT.

ASSISTANT-EDITOR:

L. M. GRIFFITHS.

SURGEON GENL'S OFFICE
LIBRARY.

"Scire est nescire, nisi id me

Scire alius sciret."

BRISTOL: J. W. ARROWSMITH;
LONDON: J. & A. CHURCHILL, 11 NEW BURLINGTON STREET.

*Price One Shilling and Sixpence.*

